# Supplementary material for: Trace Organic Compound Removal from Wastewater Reverse-Osmosis Concentrate by Advanced Oxidation Processes with UV/O3/H2O2
Source: Materials (Basel). 2020 Jun 19;13(12):2785. doi: 10.3390/ma13122785 (PMC7345651; doi:10.3390/ma13122785)
Supplement: Supplementary file 1 [file materials-13-02785-s001.pdf]

materials

MDPI

Article

Trace Organic Compound Removal from Wastewater Reverse-Osmosis Concentrate by Advanced Oxidation Processes with $UV/O_3/H_2O_2$

Aviv Kaplan [1], Hadas Mamane [2], Yaal Lester [3], and Dror Avisar [1,*]

[1] The Water Research Center, Porter School for Environment and Earth Sciences, Faculty of Exact Sciences, Tel Aviv University, Tel Aviv 69978, Israel; avivkaplan@tauex.tau.ac.il

[2] School of Mechanical Engineering, Faculty of Engineering, Tel Aviv University, Tel Aviv 69978, Israel; hadasmg@tauex.tau.ac.il

[3] Environmental Technologies, Department of Advanced Materials, Azrieli College of Engineering, Jerusalem 9103501, Israel; lester.yaal@gmail.com

* Correspondence: droravi@tauex.tau.ac.il; Tel.: +972-3-6405534

Received: 10 May 2020; Accepted: 16 June 2020; Published: date

Equation S1. – Determination protocol of the scavenging rate K_{SC}.

Pseudo-first order degradation rate constant of para-chlorobenzoic acid (pCBA) at the steady state concentration of OH·, In the case of low pCBA concentration, where its contribution to the total scavenging is negligible:

$$\frac{R^{form}}{K'} = A = \pi r^2 \frac{K_{SC}}{K_{OH,pCBA}} + \frac{K_{OH,H2O2}[H2O2]}{K_{OH,pCBA}} \quad (S1)$$

K_{SC} - scavenging rate of the water matrix [1/s]; R^{form} - Rate of formation for the OH· under the specific conditions and it is depending on the Ultraviolet (UV) lamp intensity and on the hydrogen peroxide added concentration $[H_2O_2]$; $K_{OH,H2O2}$, $K_{OH,pCBA}$ - Second-order rate constants H_2O_2 and pCBA, respectively, with OH·, where $K_{OH,pCBA} = 5 \times 10^9$ M S^{-1}.; K' - slope obtained for the log transformation of pCBA concentrations, from each set of UV applied with different H_2O_2 concentrations.

UV/H_2O_2 degradation of pCBA was examined at different H_2O_2 concentrations, and the results were used to plot $[H_2O_2]$ vs. $\frac{R^{form}}{K'}$. In other words, the equation could be describe as a linear regression, y=ax + b, where **y** is $\frac{R^{form}}{K'}$, **x** is the hydrogen peroxide concentration $[H_2O_2]$, **a** (slope) is $\frac{K_{OH,H2O2}}{K_{OH,pCBA}}$ and **b** (intercept) is $\frac{K_{SC}}{K_{OH,pCBA}}$.

K_{SC} could be calculated by extracting the intercept (b) from the linear regression equation.

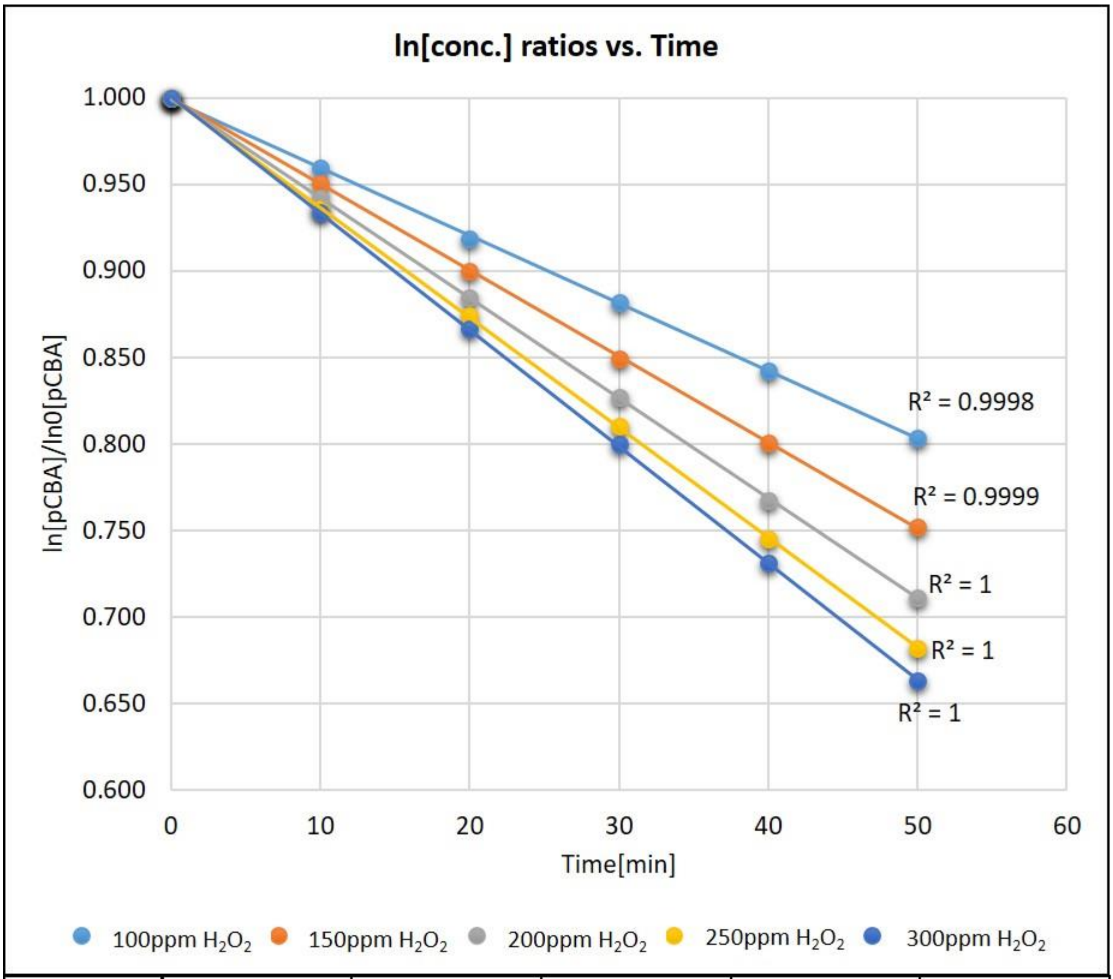

	100ppm H2O2	150ppm H2O2	200ppm H2O2	250ppm H2O2	300ppm H2O2
Slope=K'	-0.003925	-0.004977	-0.005785	-0.006363	-0.006746
Abs K'	0.003925	0.004977	0.005785	0.006363	0.006746

Figure S1. -K' calculations.

Equation S2. - R^{form} calculations protocol.

$$R^{form} = \sum_{200-300} \frac{10^{-3} \times E_p^0(\lambda)\varepsilon(\lambda)\left[1-10^{-a(\lambda)z}\right]}{a(\lambda)z} \times \varphi \times [H_2O_2] \quad \text{(S2)}$$

E^0_p – incident photon irradiance (Es cm^{-1} s^{-1}); ε(λ) - fraction of UV energy absorbed by H_2O_2 at each wavelength (M^{-1} cm^{-1}); a(λ) - solution absorption coefficient (cm^{-1}); z – depth of solution (cm); φ – quantum yield of OH· by H_2O_2 UV photolysis (mol Es^{-1});

Table 1. - R^{form} calculations based on UV measurements of the different H_2O_2 concentrations in the WWROC samples after UV radiation.

H_2O_2[ppm]	H_2O_2 [M]	R^{form}
100	$2.94x10^{-3}$	$1.52x10^{-8}$
150	$4.41x10^{-3}$	$2.22x10^{-8}$
200	$5.88x10^{-3}$	$2.91x10^{-8}$
250	$7.35x10^{-3}$	$3.58x10^{-8}$
300	$8.82x10^{-3}$	$4.32x10^{-8}$

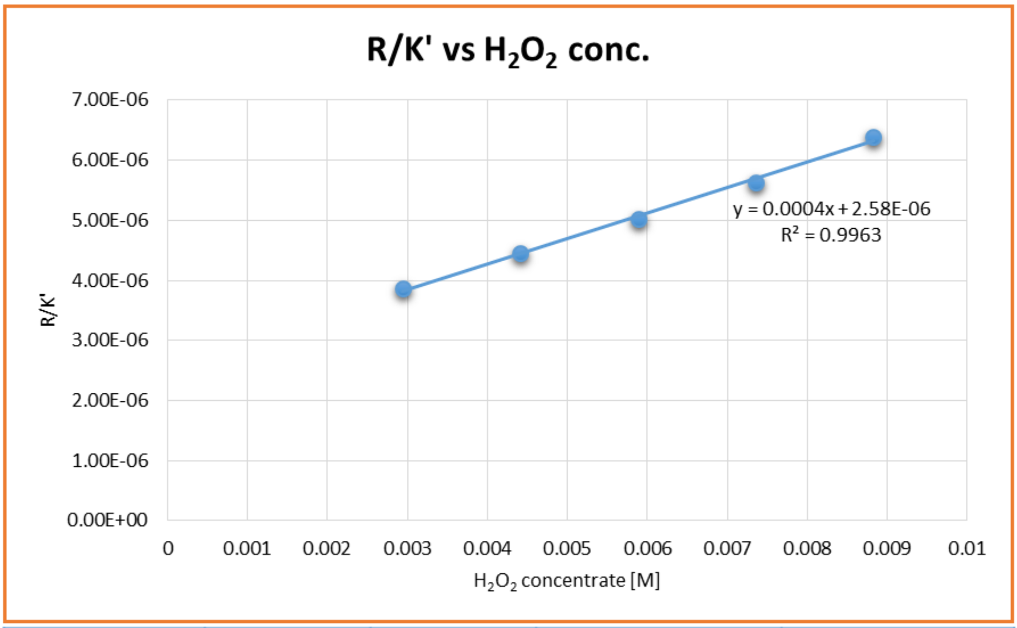

H_2O_2 [ppm]	H_2O_2[M]	(K')	R^{form}	R/K'
100	2.94E-03	3.93E-03	1.52E-08	3.87E-06
150	4.41E-03	4.98E-03	2.22E-08	4.45E-06
200	5.88E-03	5.79E-03	2.91E-08	5.03E-06
250	7.35E-03	6.36E-03	3.58E-08	5.63E-06
300	8.82E-03	6.75E-03	4.32E-08	6.40E-06

Figure S2. –plotting $\frac{R^{form}}{K'}$ as a function of the [H_2O_2]. The derived linear equation intercept is multiplied by $K_{OH,pCBA}$=5 × 10^9 M^{-1} S^{-1} to calculate the scavenging rate K_{SC}.

© 2020 by the authors. Submitted for possible open access publication under the terms and conditions of the Creative Commons Attribution (CC BY) license (http://creativecommons.org/licenses/by/4.0/).
